# Community-Dwelling Filipino Older Adults’ Experiences with Virtual Coach for Health-Enhancing Physical Activity (HEPA): A Phenomenology

**DOI:** 10.3390/nursrep15020049

**Published:** 2025-01-31

**Authors:** Michael Joseph S. Dino, Kenneth W. Dion, Peter M. Abadir, Chakra Budhathoki, Patrick Tracy Balbin, Ma. Kristina G. Malacas, Rommel P. Hernandez, Jacquelyn Joyce G. Nicolas, Jhal Barcial-Espinosa, Cheryl R. Dennison Himmelfarb, Patricia M. Davidson, Ladda Thiamwong

**Affiliations:** 1 School of Nursing, Johns Hopkins University, Baltimore, MD 21218, USA; k.dion@sigmanursing.org (K.W.D.); cbudhat1@jhu.edu (C.B.); chimmelfarb@jhu.edu (C.R.D.H.); patricia.davidson1@unsw.edu.au (P.M.D.); 2 Research Development and Innovation Center, Our Lady of Fatima University, Valenzuela City 1440, Philippines; pbalbin@fatima.edu.ph (P.T.B.); mgmalacas@fatima.edu.ph (M.K.G.M.); rphernandez@fatima.edu.ph (R.P.H.); jgnicolas@fatima.edu.ph (J.J.G.N.); jbespinosa@fatima.edu.ph (J.B.-E.); 3 College of Nursing, University of Central Florida, Orlando, FL 32816, USA; ladda.thiamwong@ucf.edu; 4 Sigma Theta Tau, International Honor Society in Nursing, Indianapolis, IN 46220, USA; 5 School of Medicine, Johns Hopkins University, Baltimore, MD 21218, USA; pabadir1@jhmi.edu; 6 The Graduate School, University of Santo Tomas, España Blvd., Sampaloc, Manila 1008, Philippines; 7 Division of the Vice-Chancellor and President, University of New South Wales, Sydney, NSW 2052, Australia

**Keywords:** mixed reality, virtual human, physical exercise, older adults, nursing

## Abstract

**Background:** Physical inactivity among older adults persists as one of the global burdens. Older adults’ physical activity (PA) levels decline simultaneously with age, causing health problems and poor outcomes. Immersive interventions, such as mixed reality virtual coaches, are gaining the potential to provide innovative solutions to prevent physical inactivity among older adults. However, an in-depth understanding of older adults’ experience in using virtual-coach-driven physical exercise programs remains unexplored. **Purpose:** This study explored the experiences of community-dwelling older adults in using a virtual-coach-driven physical exercise program. **Design:** This study applied a phenomenological design using the qualitative approach to determine the lived experiences of community-dwelling older adults about the mother project, the “Move and Groove for Older Adults Program” (Hataw at Sigla para kay Lolo at Lola), a virtual-coach-driven physical exercise program using MR. A semi-structured interview guide was crafted consisting of three domains (acceptability, barriers, and facilitators), each with two open-ended questions. MAXQDA was used to thematically analyze the qualitative data. **Results:** Nine key informants shared their experiences with the virtual coach-driven physical exercise program using mixed reality. Four themes were identified describing their experiences with the program: (1) “Looking around”: Exploring MR technology, (2) “Looking after”: Engaging with MR exercise peers, (3) “Looking at”: Examining issues with MR technology devices, and (4) “Looking Ahead”: Envisioning the future of MR exercises. **Conclusions:** The findings of this study introduced new concepts and challenged old notions regarding older adults’ technological engagement. The virtual-coach-driven physical exercise program enabled the participants to explore the novel methodology, enhance peer relationships, examine potential issues, and envision a future of possibilities using the technology. These outcomes are pertinent for progressing studies, scholars, and technology developers in incorporating MR into gerontechnology for wellness and fullness of life in the aging population. The mother project of this study was retrospectively registered [ID NCT06136468].

## 1. Introduction

The rapid aging of the population has become a global phenomenon. Currently, Southeast Asia’s aging population trends remain on a steady slope. In the Philippines, statistics predict that by the end of 2030, older adults will account for 10.4% of the general population [1,2], categorizing the country as aging [3]. Aging is universally coded as pervasive and inevitably shaped by a continuous decline in physiological function, mobility, and functional self-sufficiency [4,5]. Healthy aging encompasses measures of optimizing older adults’ physical, social, and mental function to maintain their active role in society despite the physiological changes that come with aging [6].

Older adults’ physical activity (PA) levels decline simultaneously with age [7,8]. Physical inactivity persists as the fourth leading risk factor in the global mortality rate [9,10,11]. The recent COVID-19 pandemic further aggravated this concern as studies have shown that increasing numbers of older adults do not meet the recommended physical activity guidelines [9,12,13,14]. Physical inactivity is associated with frailty, diminished quality of life [10,15], and increased risk of various chronic diseases (e.g., cardiovascular diseases, obesity, osteoporosis, depression) [16].

The World Health Organization [17] has recently updated the recommended health-enhancing physical activity (HEPA) guidelines for older adults, ranging from moderate- to vigorous-intensity physical activity with varying time allotments for a weekly duration. The integration of HEPA programs is beneficial in the prevention and management of chronic conditions, particularly in situations where no effective drug therapy is available [12,18,19,20], and in the improvement in the frailty and functional capacity of aging individuals [4]. The advantages of HEPA in the graying population are long stipulated as critical contributors to an active and healthier aging trajectory. Besides enhancing the quality of life and overall functioning of healthy and frail older adults, it was also identified that meeting the HEPA qualifications can lead to better resilience, independence, reduced impairment, improved affect and outlook in life, and extended life expectancy [9,21,22,23]. Older adults and chronically diagnosed individuals benefit primarily from physical activity as they can reap almost immediate health benefits by merely pursuing an active and healthy lifestyle [4,10].

Challenges in compliance with HEPA recommendations also persist in this specific demographic. The lack of proper and motivating strategies regarding these guidelines is at the forefront of hindering adherence [13,18]. With an optimistic outlook, introducing alternative forms of physical exercise and incorporating technology to promote compliance resulted in increased adherence and motivation toward exercise [18,24,25,26,27,28,29].

Current technologies can potentially reimagine a healthy lifestyle in the modern era, although they were previously known for being consistently linked to physical inactivity [30,31]. The recent advent of extended reality (XR) technology introduced this potential by presenting a variety of applications, such as mixed reality (MR), virtual reality (VR), and augmented reality (AR), that have congruent effects in promoting physical activity engagement and satisfaction [32,33,34,35,36]. Novel methodologies using MR and VR technology introduced optimistic outcomes in improving physical, psychological, and social functions [36,37,38]. Incorporating these emerging technologies into physical intervention programs was recently initiated, given its extensive list of advantages. The assessment of individual adherence and completion to physical exercise became easily feasible with emerging technology-based exercise programs as they can accurately monitor physical activity and deliver individualized, real-time feedback and instructions [24,29,39,40,41,42,43].

Virtual coaching systems also became prominent with the emergence of virtual coaches, or e-coaches, that can provide personalized support and almost mimic human coaches in delivering health interventions with measurable improvements in physical activity [30,44,45,46]. The study of Dino et al. [47] clearly shows that using virtual coaches in community-based HEPA programs among older adults is comparable to traditional methods. Virtual coaches also produced promising results in increasing the quality of life and decreasing the healthcare needs of older adults [48], as seen through literature reviews [30] and experimental studies [44,45]. There are also existing protocols that examine the potential of MR in HEPA within the context of developing nations [49]. However, recommendations on older adults’ use of virtual coaches in physical exercise programs remain relevant, with existing studies proving that this methodology is still in its infancy.

A qualitative investigation of the factors that promote and inhibit older adult engagement in a virtual-coach-driven HEPA program remains relevant. Given the abundant literature on the quantitative aspects of virtual coaches in technology-assisted HEPA programs for older adults, there remains a minute amount of literature exploring the phenomenological inquiry of this novel methodology.

To supplement this gap, this research shall tap into the lived experiences of community-dwelling older adults exposed to a virtual coach-assisted HEPA program. This study aims to provide a detailed description of the experiences of community-dwelling older adults in the “Move and Groove for Older Adults Program” (Hataw at Sigla para kay Lolo at Lola), which is a virtual-coach-driven physical exercise program using MR. Older adults in this study are posited to elicit significant experiential insights about this phenomenon.

## 2. Review of the Literature

### 2.1. Technology-Driven Health-Enhancing Physical Activity

The association between physical inactivity and technology-based programs has become prominent in contemporary society [31]. This phenomenon thereby profoundly impacts the health of individuals all around the world. Moreover, factors causing a sedentary lifestyle, such as low participation and compliance with physical activity programs, are primarily linked to limited healthcare access and patient-specific barriers [50,51]. This calls for redefining the role of technology in countering inactive lifestyles. Several technological applications have been identified in the current literature that have shown the ability to promote interest and compliance [52], as demonstrated by the use of physical exercise programs. Examples of these applications include the use of videoconferencing platforms [53,54], exergaming devices [55,56], and health-related applications installed on personal digital devices [57,58]. Moreover, a study [59] explored novel solutions to these barriers using home-based fitness regimens and technologies. Group exercises undertaken through teleconferencing platforms (e.g., Zoom) and exergaming applications have been shown to have high adherence rates, satisfaction, feasibility, and adaptability [51], contributing to beneficial medical effects, including weight loss [60], better sleep quality [61], and pronounced glycemic and blood pressure control [62,63].

Emerging technology also has the potential to introduce novel methodologies that can promote physical activity among the population. Considering the significance of its expansive relevance and benefits to physical activity and health, Margrett et al. [64] redefined the trajectory of conventional exercises in response to existing research on the potential for XR, including VR, MR, or AR. Few studies have been conducted to maximize this advanced technology’s potential in enhancing health programs. Exercise regimens designed as a game in an MR environment using a head-mounted display (exergaming) yielded considerable outcomes regarding user performance, biofeedback response, and engagement [65]. On the one hand, Kaplan et al. [66] corroborated the conclusion that using MR in physical fitness programs is as successful as traditionally acknowledged approaches, especially when traditional methods are regarded as far-fetched. Immersive VR platforms as exercise program mediums, on the other hand, have been proposed with promising results in terms of user engagement and satisfaction [67], as well as a variety of applications such as exercise therapy [68,69], pain management [70,71,72], and improved functional ability [73,74,75].

Kamali et al. [30] proposed that the innovation and diffusion of mobile technologies (i.e., smartphones and connected objects), artificial intelligence (AI), and robots ushered the path for the birth of virtual coaches, or e-coaches, capable of supporting, complementing, and potentially replacing human coaches in health interventions. Meanwhile, research on the application of AR with virtual coaches has revealed that older adults regard the methodology as encouraging and stimulating and the virtual coaches as alive, harmonic, intelligent, and pleasant humans [76,77]. Likewise, the gamification of physical activities among older persons demonstrated the significance of virtual coaches and user interaction [78]. Further, an artificially intelligent virtual assistant-led lifestyle modification intervention was feasible and achieved measurable improvements in physical activity, diet, and body composition using tailor-made circumstances that were more compelling than generic ones, and people had a positive attitude toward the virtual coach [44,45,48].

XR has been incorporated to promote HEPA. VR exergames related to HEPA have been evident to promote physical activity [79,80,81,82] alongside addressing persistent cognitive and psychological challenges, such as cognitive decline and depression among all age demographics [83,84]. Similarly, VR programs related to HEPA improve physical performance and self-efficacy in exercising, notably catering to the needs of older adults [85,86,87]. In contrast to the aforementioned utilization of AR and VR, MR persists as an uncharted field of research, especially the potential of HEPA for the community-dwelling older population. Delving into the potential of MR to promote HEPA for older adults will open up an opportunity to address persisting barriers that inhibit its use, such as usability and design issues [88,89], social stigma, acceptance of technology use [90,91], and the gap in the training and support needed to use such technology [92,93], and empower its noted benefits on older adults’ physical [94], cognitive [95], social [64], and functional health [91,96].

### 2.2. HEPA Experiences of Older Adults

The aging population in Asia–Pacific has reached an all-time high. In particular, the number of people aged 60 and above in the Philippines is projected to surge by 11.8% in 2050, up from 5.3% in 2019 [2]. The Philippine Statistics Authority (PSA) [97] denoted that individuals aged 60 belong to the vulnerable population primarily associated with various health conditions, namely immunosuppressing maladies like cancer [98], diabetes [99], and cardiorespiratory complications such as COVID-19 [100]. Moreover, Dahonan-Cuyacot et al. [101] claimed that the Philippine National Health Accounts allocated PHP 171.5 billion for the older population aged 60 years and above, with about 26% designated for older populations with documented medical conditions. Hence, the Philippine government ought to establish an efficient resource allocation scheme considering the strain the country would face due to the preceding demographic transition [102]. The World Health Organization [103] confirmed that the Philippine government launched a cross-departmental framework, including several forms of HEPA activities, in response to the dramatic spike in age-related diseases. This prompted the emergence of Zumba, which is a form of physical exercise comprising an array of fitness facets to promote musculoskeletal health [104]. The cornerstone of such activity is aerobic training, thereby improving calorie consumption and significantly reinforcing the cardiovascular system as well as, to a lesser extent, anatomical systems and beyond [105]. Furthermore, most older people, notably in developing nations like the Philippines, are actively engaged in aerobic exercise programs like Zumba [106]. Still, Inouye et al. [107] remarked that a standard Zumba class comes with fast-paced routines that could not accommodate people over 65 with documented functional limitations. Because of the evident challenges regarding the musculoskeletal part of Zumba, a lower-intensity version called Zumba Gold was crafted [108]. Two feasibility studies conducted on this version of Zumba have confirmed that it is safe and pleasing for those with Parkinson’s disease [109] and suitable for hemodialysis patients [110]. Thus, the positive implications of the Zumba program on the general health of Filipino older adults, as well as its integration into other community sectors, warrant further research.

Sharma et al. [111] asserted that approximately one-third of the global adult population is physically inactive, contributing to nearly thirteen (13) million disability-adjusted life years (DALYs) and premature mortality. Considering that the elderly population becomes generally sedentary, promoting physical activity is indispensable for augmenting their health, along with its benefits in all areas of the older population’s health [112,113,114]. Moreover, the WHO [11] advocated a strategy (i.e., active societies, active environment, active people, and active systems) to reduce physical inactivity among older people by 2030. They also published a provision recommending that an older adult participate in at least 150 to 300 min of moderate-intensity physical exercise or 75 to 150 min of vigorous physical activity [17]. Physical exercise should encompass muscle development and functional balance for at least two and three days each week, respectively. HEPA has beneficially impacted physical wellness, namely cardiovascular health [115,116,117], pain reduction management [118,119], musculoskeletal strength, and balance [120,121,122], and magnified quality of life (QOL) [4,123,124]. Likewise, physical activity boosts psychological and cognitive attributes such as sound mental health [125], mood enhancement [126], depressive symptomatology [127], and working memory [128]. However, there frequently exist constraints to immersing the older population in a physically active lifestyle, such as pre-existing health conditions [129], indolent behavior [130], inadequate professional guidance and social support [9,131], and inaccessibility of HEPA options and program-related information [132]. The study of M. Chen et al. [133] states that early detection of the aforementioned constraints has profound advantages in averting detrimental effects on the functional skills of the ever-growing older adult population who lead inactive lifestyles. The prevalence of high BMI measures (i.e., overweight and obesity), existing medical issues, and poor mental and physical health are among the behavioral characteristics to be assessed in configuring interventions to satisfy older adults’ specific needs. This will result in developing a policy for such demographics that promotes prompt HEPA adherence while providing the requisite social and emotional support.

## 3. Methods

### 3.1. Design

A constructivist phenomenological design [134] under the qualitative approach was applied in this study to determine the lived experiences of community-dwelling older adults about the mother project, the “Move and Groove for Older Adults Program” (Hataw at Sigla para kay Lolo at Lola), which is a virtual-coach-driven physical exercise program using MR. Phenomenology was the most suitable for gaining insight into the experiences of older adults in the program. Several studies have also utilized this design in determining the experiences of older adults [135,136] in technology usage [137,138] and physical activities [52,139].

### 3.2. Rigor and Reflexivity

The chosen design and paradigm align with this study’s aim to explore the lived experiences of older adults participating in the technology-driven HEPA. Through this design, the researchers ensured an in-depth exploration of participants’ subjective experiences. The choice of sampling method ensures that the nine physically able older adults gained substantive and pertinent experiences from the program. The screening conducted by board-certified gerontologists added rigor to this study, ensuring that the participants met the health and study criteria. The semi-structured interview guide was thoroughly crafted and delivered in the Filipino language to cover three essential elements: acceptability, barriers, and facilitators, alongside preliminary and closing questions to engage participants and foster a more profound understanding. The transcriptions and participant validation were verified to enhance credibility and ensure that the findings genuinely mirror the participants’ lived experiences. The comprehensive description of the methodology also provides transparency, allowing readers to evaluate this study’s processes and results critically.

### 3.3. Key Informants Selection

**Figure 1:**
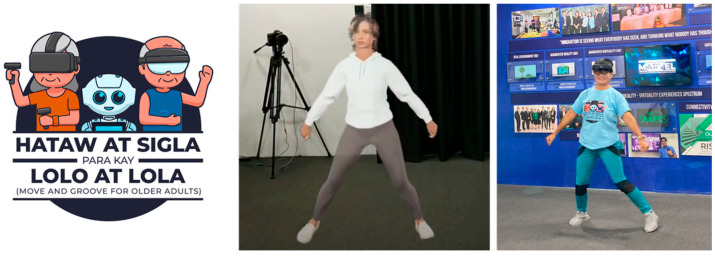
Health-enhancing physical activity using virtual coach projected via mixed reality.

Nine (9) [140] purposively selected community-dwelling older adults from two senior centers in Metro Manila, Philippines, served as key informants in this study. Physically able older adults with rich experiences who have completed and actively participated in the mixed reality virtual-coach-driven health-enhancing physical activity project, the “Move and Groove for Older Adults Program” (Hataw at Sigla para kay Lolo at Lola; Figure 1), were chosen to be interviewed. All participants were (1) within the age range of 60 to 75 years old, (2) ambulatory, (3) able to comprehend basic directions, (4) had normal visual acuity (those with corrective eyewear are allowable), and (5) willing to participate in the program, provided that they signed an informed consent. Board-certified gerontologists accomplished health and program records screening before capturing the data to ensure safety and compliance with the criteria.

### 3.4. Qualitative Tools

The semi-structured interview guide (Table 1) was carefully crafted to correspond with the experiences of older adults on the program. The guide consists of three domains: acceptability, barriers, and facilitators, each with two open-ended questions. The acceptability domain consists of questions that explore their acceptance of the virtual coach and their opinions on the program. The second domain, barrier, delves into the problems they encountered during the program, such as health issues and the aspects they want to change. Lastly, the facilitator domain refers to the older adults’ suggestions to improve the program and technology, as well as ways of enticing others to partake. In addition, there were three preliminary questions and two closing questions to increase rapport with the respondents and comprehensively conclude the interview, respectively. The tool was carried out in the Filipino language.

### 3.5. Data Gathering Procedure

The mother project, the “Move and Groove for Older Adults” project, involved the development of a mixed reality virtual coach system using the Mixed Reality Toolkit version 2, Unity 2018.4.x, and Unreal Engine version 5 software. The development of the virtual coach was in accordance with the results of a previous conjoint study, which followed a human-centered technology development approach [141]. Motive: Body^®^ was utilized to produce human-like movements and expressions in the developed virtual coach, while GarageBand^®^ was used for incorporating music in the program.

The developed virtual coach was employed through the Optical See-through Head-mounted Display device during the participants’ exercise sessions, which lasted 1.5 h each. Each session comprised an orientation, warm-up, and stretching before the mixed-reality-driven exercise. The program lasted for a month, with two sessions each in the initial two weeks and three sessions each in the final two weeks. During the exercise sessions, a participant only followed the virtual coach through the OST-HMD.

The occurrence of cybersickness during the exercise sessions was not evident across all participants; however, one participant mentioned that they experienced headaches while wearing the OST-HMD device due to its heaviness. Breaks were implemented in any occurrence of adverse effects, and the continuation of the exercise session was upon the recommendation of the gerontologist present.

At the conclusion of the mother project, two phases of data collection were accomplished:

Step 1: Preparatory. Preparation is essential for phenomenological research [142]. This includes visiting two senior centers to obtain permission, proceeding with the orientation, and purposively selecting the nine respondents during December 2023. In selecting the respondents, their health and program records were carefully screened to ensure their compliance with the criteria.

Step 2: Interviews. The interviews were conducted on the last day of the program and carried out singly and separately in three private and quiet rooms, with one interviewer per room. The respondents were only required to attend one session at their convenience. The older adults were initially reminded about the purpose of the interview and consented to allow audio recording. They were allowed to choose which question to respond to and not to, without any consequences. The in-depth interviews lasted 30 to 50 min, depending on the length of the responses. To ensure credibility, the interviews were allowed to be lengthy to extract all possible answers, and observations were recorded in a journal [143].

### 3.6. Data Analysis

All audio recordings of the interview were transcribed in Microsoft Word. To ensure the accuracy of the transcription, the recordings were listened to three times [144]. The documents were then uploaded to MAXQDA for processing. Open-ended coding was performed, and themes were generated, guided by the central question of this study. Key informant validation was conducted to verify the accuracy of the themes.

### 3.7. Ethics

This study was granted ethical clearances by the Johns Hopkins Institutional Review Board and Our Lady of Fatima University Institutional Review Board under reference numbers IRB00347131 and 2022-IERC1-20299V2-01, respectively. The mother project was retrospectively registered at ClinicalTrials.gov [ID NCT06136468]. All the study participants provided their written informed consent.

## 4. Findings

**Figure 2:**
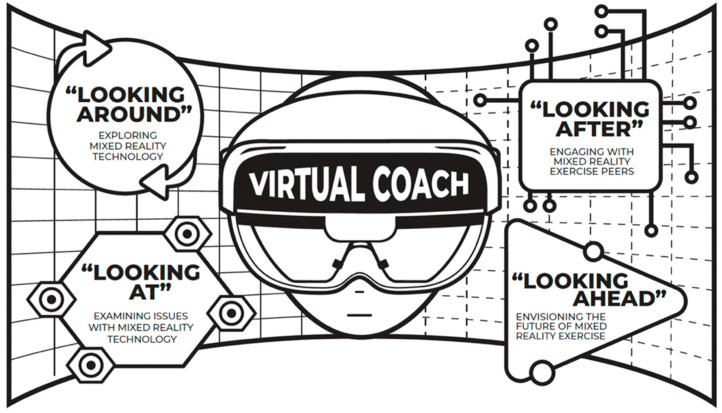
Themes defining older adults’ experiences with the mixed reality virtual coach.

Nine key informants shared their experiences with the virtual coach-driven physical exercise program using mixed reality. Four themes were identified describing their experiences with the program: (1) “Looking around”: Exploring MR technology, (2) “Looking after”: Engaging with MR exercise peers, (3) “Looking at”: Examining issues with MR technology devices, and (4) “Looking Ahead”: Envisioning the future of MR exercises. The thematic embodiment is showcased in Figure 2 and Table 2.

Theme 1—“Looking around”: Exploring mixed reality technology.

The theme “Looking around”: Exploring mixed reality technology relates to the experiences of the key informants in exploring the mixed reality virtual coach technology. The older adults narrated their joy and excitement in using the technology. Although some perceived the technology as for younger generations and similar to computer games, they gained confidence in using it.

Theme 2—“Looking after”: Engaging with mixed reality exercise peers

For the older adult participants, the program serves as a channel to socialize and connect with others, thus the theme: “Looking after”: Engaging with mixed reality exercise peers. They gained new friends and felt a sense of belonging as part of the group.

Theme 3—“Looking at”: Examining issues with mixed reality technology devices

The key informants also verbalized some issues for improving mixed reality technology. Some participants consider the weight as a pull factor in using the device. Some non-digital-native older adults experienced difficulty navigating the system, while others experienced sensory challenges. These concerns were tagged under “Looking at”: Examining issues with mixed reality technology devices.

Theme 4—“Looking Ahead”: Envisioning the future of mixed reality exercises

The key informants communicated several possibilities and future technology applications. They consider the mixed reality virtual coach as a mobile system for performing physical exercises at home. Other participants suggested several options to be added to the device. Ultimately, most key informants are looking forward to using the technology soon.

## 5. Discussion

This study examined using MR virtual coaches for HEPA among older adults. Multiple themes were generated from the key informants’ physical exercise experience with a virtual coach: (a) Looking Around, (b) Looking After, (c) Looking At, and (d) Looking Ahead. These themes relate to the robustness and complexity of human experiences from exposure to a novel methodology [145]. Traditional exercises and technology-driven HEPA may share the same goal of limiting physical inactivity, but these methods may differ from each other in terms of participants’ experiences [146], as shown in the results.

The first theme is “Looking Around”. The participants shared their perceptions of exploring the technology, the system behind it, and the benefits. The program enabled the participants to gain confidence in exploring MR technology despite initially perceiving it as only for the younger generation. Regardless of the unfamiliarity with the technology, the participants still enjoyed and behaved naturally and were interested in trying it again [147]. This also shows that the assistance of a virtual coach provides significant value to older adults using the MR. This result is in contrast to previous studies implying that older adults have difficulty using technology due to inaccessibility, disinterest, and the presence of physical impairments [148]. One study also shows that older adults have different perceptions of current technologies and their usage [149]. Moreover, the involvement of older adults in technology enhances and maintains a healthier lifestyle and gives them security [150]. As technology innovates further, the demand and accessibility it holds also increases. Technological advancements help older adults, suggesting that these emerging technologies were not limited to the current generation but also beneficial to the older ones.

The second theme is “Looking After”. The program allowed participants to experience shared camaraderie and mutual understanding among each other. The participants felt a sense of belonging and gained new peers. This theme opposes the predominantly accepted truth that technology promotes social isolation [151,152]. Findings from this study have shown that technology can promote meaningful participant relationships through shared interests and activities, concluding that technology can cause social relations instead of isolation. Similar studies have shown that long-term use of technology can encourage a sense of companionship in older adults’ daily activities [153] and downplay isolation in this cohort [154,155]. The integration of digital technology in geriatric facility interventions has become more frequently employed to decrease the sense of isolation in older adults [156] given its potential to provide fulfilling and gratifying relations even in a contactless, long-distance situation [157,158]. Therefore, it cannot be denied that technology can foster social collaboration and engagement [121,159,160], making technology a good strategy for fundamentally solving social isolation for older adults and anyone who uses it [161].

The third theme is “Looking At”. Participants have identified the positive and negative effects of technology after exposure to virtual-coach-driven physical exercise. This study’s findings show that despite the perceived flaws of technology, the participants regarded it as useful and, given its novelty, remained curious and looked forward to it. It was also found that some participants may have been receptive to technology because it was not fully apparent and utilized during their time [162]. The participants also articulated some issues and challenges using the technology. Non-familiarity and nonacceptance resulted in difficulties and resistance to technology-driven interventions among older adults [163]. Acceptance of digital technology is often hindered by mistrust and distrust regarding security issues and information disparities [164], resulting in a phenomenon called *Technophobia*, where older adults feel reluctant to use technology due to computer anxiety and technology usability [165]. However, considering factors such as the usefulness of technology, trust, data quality, and shared responsibility between users and providers facilitates increased participation among older adults in technology-assisted interventions [166]. Therefore, immersing older adults in technology using appropriate strategies might result in them having a better experience utilizing it. This may be the head start for policymakers to craft more technology security and safety policies, emphasizing older adults’ digital needs and preferences. Technological improvements are necessary to promote willingness, acceptance, and safety tailored to older adults’ holistic well-being. This may include appropriating the weight and usability of digital devices, making them comfortable for more dynamic movement, and safer for older adults who are visually impaired or have sensory troubles [162,167,168].

Lastly, the fourth theme is “Looking Ahead”. After being exposed to technology, older adults gained the momentum of becoming potential digital users. The findings substantiated the participants’ optimistic outlook on the future of XR technology. The conducted program encouraged the participants to envision a future filled with opportunities using technology. The forthcoming industrial revolution might usher in countless applications of XR technology, particularly MR, especially for older adults, which encompasses both dynamic and diurnal activities [35,78,138,169]. MR could become the future of the digital world due to its promising outcomes in aiding humans and making technology more convenient and accessible [170,171]. Notably, due to the recent COVID-19 pandemic, technological interventions to enhance physical and cognitive functions in the older population are gaining momentum [172,173,174]. Technology holds a significant possibility in improving older adults’ quality of life, giving rise to the endless potential for healthy aging [6,30,48,175].

## 6. Conclusions

A qualitative investigation of the factors that promote and hinder older adult engagement in a virtual-coach-driven HEPA program remains relevant. This study aims to describe the experiences of community-dwelling older adults in a physical exercise program using a virtual coach. Multiple themes were identified from the participants’ robust and rich experiences. The program enabled the participants to explore the novel methodology, enhance peer relationships, examine potential issues, and envision a future of possibilities using the technology. The findings of this phenomenological inquiry must be acknowledged in parallel with some of the limitations present. The research included a limited sample of purposively selected older adults from two senior centers in a developing country. Subsequent studies may wish to consider expanding the study sample and exploring other experiential aspects of technology use among older adults.

Based on the outcomes of this study, fourfold implications are evident. To begin with, the outcomes show the complexity of the experience of technology utilization, especially for older adults, which exhibited diversity. Future research must focus on developing user-centered technologies, including MR, that demonstrate inclusivity in relation to age. Next, the results deviate from the convention that technology causes social isolation. Scholars must explore the extent to which emerging technologies promote social collaboration. Following that, technology developers must work to mitigate the negative effects of MR technologies, especially on older adults. Subsequent studies must investigate ways of adapting technologies based on the capabilities of older adults. Finally, this growing population is a potential user of technology in the foreseeable future. Further investigations must incorporate MR into gerontechnology to promote wellness and fullness of life in the aging population.

## Figures and Tables

**Table 1:** Open-ended questions in the interview guide.

A. Acceptability
1. Have you liked the program? Can you specify what parts you have really liked?
2. What is your opinion on the technology that you have used?
B. Barriers
1. Have you encountered any problems with the program? Are there any parts of the program that were hard for you?
2. Do you want to change any aspect of the program? How about the technology?
C. Facilitators
1. Do you have any suggestions to improve the program and make it more appealing to older adults?
2. What are the things that can attract you to join the program in the coming years?

**Table 2:** Summary of themes related to key informants’ exercise experiences with virtual coach technology.

**Theme**	**Description**	**Representative Transcript Excerpts**
“Looking around”: Exploring mixed reality technology	The program enabled the participants to explore mixed reality technology.	▪ I like it better than watching on YouTube. I love 3D. I like games. [CG11] ▪ We are proud to be the first ones to experience this exercise technology. [CG11] ▪ I like the option of selecting the coach and music. It provides variety. I like the many dance steps. It’s not boring. [CG11] ▪ We enjoyed everything. It makes us hooked to the program. [CG18] ▪ I am proud of what I am doing. It makes me feel happy and strong. We haven’t had anything like this before. I like to move. [CG18] ▪ I feel better about myself. I can feel my improvement. [CG18] • It takes time to cope with the exercises. But I must start somewhere before I can perfect everything. [CG19] • Seeing the virtual coach makes me feel younger. It’s similar to a computer game. [CG158] • I enjoyed the program and am learning many ways to exercise and try different technologies. I also gained confidence. I am looking forward to the next schedule. [CG33] • Using the technology makes me think. It exercises my brain and body. It releases a lot of stress. I wish I had more time with it. [CG33] • It is exercise gamified. I feel younger. My grandkids will love it. It’s a different experience. • I prefer the female virtual coach. I think I can relate to her very well. [CG33] • I feel proud that I tried this technology. For me, it changes my perspectives about technology and exercise. [CG32]
“Looking after”: Engaging with mixed reality exercise peers	The program enhanced the participants’ relationships with their peers.	▪ It would be more exciting if we used the technology together. It makes us gel with one another—trying new things. I am looking forward to every session. It’s like studying again. [CG11] ▪ I hope you will offer the program to others, too. I am sure they will enjoy the program. [CG11] ▪ My friends and I are chatting about the program. We maintain a group chat. We enjoyed sharing what we experienced. We love to wear matching outfits. We love being with one another. [CG18] ▪ I love to be part of the group. We meet outside the program schedule. We check on each other. We might not know each other’s names, but I know their faces. [CG18] ▪ I appreciate this program. I learned a lot. I gained friends. [CG158] ▪ I enjoyed the company of my peers. We meet outside the exercise schedule and talk about things. [CG157] ▪ We [seniors] are happy together. It makes the feeling better and lighter. [CG32]
“Looking at”: Examining issues with mixed reality technology devices	The program empowered the participants to examine potential issues with technology.	▪ I am curious about the price of the device. I hope I can afford it. [CG11] ▪ The device is quite heavy. If you wear glasses, it’s not comfortable. For me, I need to wear my glasses. [CG11] ▪ You need to look straight ahead to see the virtual coach. If you move sidewards, it will not move. It stimulates your senses. [CG11] ▪ The technology seems expensive. [CG11] ▪ It slips in my head sometimes because I have a small head. I’m doing my best to adjust. [CG18] ▪ I have difficulties initially with the controls. But it’s all worth it. [CG18] ▪ We are discussing it [technology]. We feel it’s expensive. We might not afford it. [CG18] ▪ It’s quite heavy, and I had headaches. Are these technologies for youngsters? I can’t operate computers very well. [CG22] ▪ I can’t see the coach very well without glasses. It’s blurred. [CG32]
“Looking Ahead”: Envisioning the future of mixed reality exercises	The program engaged the participants to reimagine a future of possibilities with mixed reality technology.	▪ The virtual coach device can be used even at home. For example, when the weather is not good. You can do exercise at home. [CG11] ▪ The virtual coach is also applicable to working seniors. For instance, if you have a business that you need to attend to. You can use it anytime based on your preferences and schedule. You can use it at home. [CG11] ▪ I know I have made a lot of progress. I can feel it. It is my personal goal to be healthy. I want to join the next batch. [CG157] ▪ I enjoyed the music; it fits our generation. I wish there would be remixes. ▪ Hopefully, it will be more colorful. And, can we play sports in it in the future? Like badminton and tennis? [CG33]

## Data Availability

The raw data supporting the conclusions of this article will be made available by the authors on request.

## Public Involvement Statement

No public involvement in any aspect of this research.

## Guidelines and Standards Statement

This manuscript was drafted in line with the standards for reporting qualitative research: a synthesis of recommendations (SRQR) for qualitative research.
